# Impact of Fabrication Defects on FPGA Logic Using Memristor-Based Memory Cells

**DOI:** 10.3390/mi17040429

**Published:** 2026-03-31

**Authors:** Jonas Schoenen, Jonas Gehrunger, Leon Mayrhofer, Timo Oster, Eszter Piros, Taewook Kim, Alexey Arzumanov, Enrique Miranda, Klaus Hofmann, Lambert Alff, Christian Hochberger

**Affiliations:** 1 Computer Systems Group, Technische Universität Darmstadt, 64283 Darmstadt, Germany; 2 Integrated Electronic Systems Lab, Technische Universität Darmstadt, 64283 Darmstadt, Germany; 3 Advanced Thin Film Technology Division, Technische Universität Darmstadt, 64287 Darmstadt, Germany; 4 Departament d’Enginyeria Electrònica, Universitat Autònoma de Barcelona, 08193 Barcelona, Spain

**Keywords:** FPGA, memristor, fault-tolerance, logic synthesis

## Abstract

Memristor-based configuration memory offers an alternative solution to the volatility and large area overhead of conventional Static Random Access Memory (SRAM)-based FPGA configuration memory. Their non-volatile nature and the possibility of stacking them on top of the logic layer in a process called Back-End-Of-Line (BEOL) manufacturing help not only dramatically reduce area consumption but also significantly reduce startup time. However, due to the comparatively high defect probability caused by manufacturing defects, traditional approaches for defect tolerance are not fit to address these defects. This work introduces an approach to defect-aware and tolerant synthesis. Based on this, an investigation into the defect tolerance of different architecture choices regarding the size of LUTs and the fracturability of LUTs is presented. We can show that smaller, non-fracturable LUTs exhibit a higher defect tolerance. Moreover, multiple strategies to improve the mapping result based on the properties of the logic functions are introduced. Notably, reducing the mapping complexity of logic clusters during the packing stage significantly improves the mapping success rate.

## 1. Introduction

Field-Programmable Gate Arrays (FPGAs) have become essential tools in modern computing due to their reconfigurability, parallelism, and ability to accelerate computational workloads. The bit-level reconfigurability of FPGAs provides a cost-effective alternative for implementing specific hardware compared to Application-Specific Integrated Circuits (ASICs). However, due to the significant area overhead introduced by the necessary configuration memory and interconnect structures, the average design is 35 times larger and 4 times slower than a comparable ASIC implementation [1]. Moreover, due to the volatile nature of SRAM, which is used as configuration memory, the programmed functionality is lost when the chip is powered down.

Memristors, postulated by Leon Chua [2] in 1970 as the fourth passive electronic circuit element, promise to offer a compelling alternative to SRAM-based memory cells. After the construction of a functional memristor in 2008 [3], many different memristor implementations in various material systems have emerged. The fundamental connection made by memristors is the combination of charge and flux, leading to a circuit element which has a memory of the charge that has flowed through it in the past. With this memory, the resistance of the memristor is altered. The change of the resistance can vary between a (quasi) analog range and purely binary states depending on the underlying material. Whereas analog memristor implementations are of great interest to neuromorphic research, this paper focuses on the application potential of binary variants.

The working principles behind these memristors primarily include ion migration [4] and carrier trapping/detrapping [5]. While their switching mechanisms differ, the basic structure of these types of memristors is the same. They comprise two conducting contact plates, an initially non-conducting insulator layer between them and a charge carrier reservoir. Notably, one of the contact plates can simultaneously serve as the reservoir. In most devices, programming is done electrically. When voltage is applied to the memristor in the set direction, a conducting filament in the insulating layer forms, which lowers the resistance. If voltage is applied in the opposite direction, the filament grows back into the reservoir. The filament can be comprised of charge carriers (e.g., oxygen, hydrogen, atom vacancies) or conducting metal ions. The two resistance states attainable by binary memristors are called Low Resistive State (LRS) and High Resistive State (HRS), with analog devices additionally featuring stable intermediate states between the extremes. The behavior of the resulting memristors can be tuned depending on the intended application, trading off, e.g., the ratio between HRS and LRS, also referred to as memory window with the stability of states and the energy/voltages required to switch between them. By selecting the appropriate material and appropriate tuning, e.g., through defect engineering, stable states, switching energy and large endurance for write cycles could be achieved with very small resistance steps between separate stable states [6].

Current research shows promising retention rates and time for memristors, which makes them a suitable candidate for non-volatile memory (NVM). Therefore, the deployment in the configuration memory of FPGAs is a compelling use case for memristors, which tackles many limitations of today’s FPGAs traditionally utilizing SRAM-based memory. Due to their non-volatile nature, memristor-based NV-FPGAs do not need a reconfiguration after a power-cycle. Furthermore, many material systems for memristors like $HfO_{x}$ are CMOS process compatible. This offers the possibility of stacking memristors on top of the logic layer during back-end-of-line (BEOL) manufacturing, significantly reducing the footprint of the configuration memory [7].

However, memristor fabrication and especially BEOL manufacturing is inherently less reliable than that of SRAM cells. Particle contamination, forming variability, and material defects can render memristors defective to a degree that makes the production of entirely defect-free chips very unlikely. Today’s FPGA architectures, as well as synthesis software, such as Verilog-to-Routing (VTR) [8], are not capable of dealing with defects in the configuration memory.

In this paper, a comprehensive study on the impact of spatial defect patterns on different architectures of function-generating Look-up Tables (LUTs) and logic blocks is presented. Moreover, techniques to improve the quality of the mapping result, without altering the underlying hardware, are investigated. In this context, logic function properties that facilitate better mapping into defective LUTs are identified and discussed. This paper focuses exclusively on defects within the logic and will not discuss the impact of defects on the routing infrastructure. While defects in the interconnect will probably significantly reduce the mappability of designs and will deteriorate the mapping results, the investigation of interconnect defects requires a whole different set of tools and approaches that need to be developed first.

The remainder of this work is structured as follows: After introducing some related work (Section 2.1) and synthesis toolchains (Section 2.2), the memristor’s defect model is introduced (Section 2.3). Based on this, the mapping into defective LUTs is presented (Section 2.5), followed by a discussion of logic function properties used to improve the mapping results (Section 2.6). The Results (Section 3) presents a comprehensive study on how different architectural decisions impact mapping quality in the presence of memory cell defects (Section 3). Moreover, the discussed improvements are evaluated and the work is concluded by a discussion of the results in Section 4.

## 2. Materials and Methods

### 2.1. Related Work

The idea of using memristors as configuration memory for non-volatile FPGAs has been discussed in previous works. Ref. [9] introduces an all-new 3D FPGA architecture that stacks the memristor layer on top of the logic layer. While using the same LUT architecture and replacing only the SRAM cells with 1T2R cells, the authors propose a new routing architecture. They claim that the new architecture achieves 5.18× area savings, a 2.28× speedup, and a 1.63× power savings. Ref. [10] summarizes the advantages and challenges of implementing memristor-based configuration memory. The authors identify the initial forming, which sets up the electrical characteristics of a memristor, as well as the programming, as significant challenges when integrating memristors into a design. They argue that the required circuits can require a significant amount of chip area, potentially nullifying the area benefits of memristors. Furthermore, they conclude that the read process of memristors can discriminate the stored value, causing endurance problems. Consequently, they argue that specialized CAD toolchains are required to address these problems. However, the problem of handling a potentially significant number of defective memristors per chip is not discussed.

Other works have addressed manufacturing defect tolerance in the light of shrinking structure sizes. Ref. [11] provides a survey of multiple approaches to manufacturing defect tolerance. The survey classifies them into two categories. The first class of approaches uses device-level tolerance mechanisms. These approaches propose additional spare resources that can be used in case of an error, without involving external processing. These approaches include adding extra rows or blocks and using additional resources to enable shifting functionality from defective elements to spare ones.

The second class of approaches requires configuration-level changes, aided by external processing, while not requiring architectural-level changes to the device. Most of these approaches avoid defective elements altogether. One example is presented in [12]. The authors propose a modified mapping toolchain that is aware of defective elements and avoids them during placement and routing. The authors mark logic slices as defective with a uniform distribution of errors. If a slice is defective, it is not used. They can demonstrate that for designs with a utilization of 50% and a slice defect rate of 30%, a timing degradation below 10% can be achieved. It should be highlighted here that a slice contains many logic blocks and even more LUTs.

A more sophisticated approach is proposed in [13]. The authors modify an existing mapping to address defective memory cells by using spare LUT inputs, spare LUTs within logic blocks, and local rerouting to avoid defects in the global routing infrastructure. To reduce the time required to modify the mapping, the use of spare LUTs during the initial mapping was also investigated. However, the authors considered only tolerance to single defects.

On the manufacturing side, a lot of work has gone into manufacturing memristors and characterizing typical defects that occur during BEOL manufacturing. Ref. [14] shows different manufacturing defects resulting from small to large particle contaminations and material defects. Refs. [15,16] study different defects that occur specifically during BEOL manufacturing. They describe how such defects occur and what impacts they have. For this work, the permanent Stuck-at-Faults (S@), caused by variations in the Forming process and through contamination, are particularly interesting.

### 2.2. Logic Synthesis Toolchain

**Figure 1:**
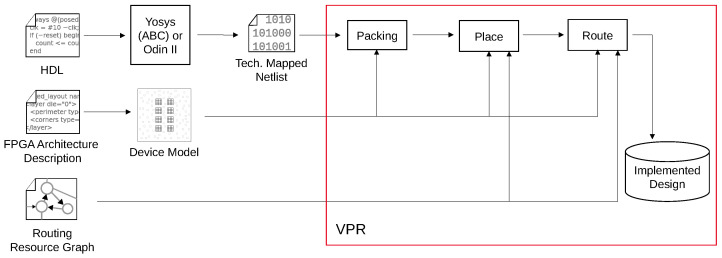
Synthesis flow for FPGAs using VTR/VPR, with steps performed by VPR indicated with a red outline.

The process of mapping a design into the structures provided by an FPGA is performed by a logic synthesis toolchain, such as the open-source toolchain Verilog-to-Routing (VTR) [8]. VTR takes in a design implemented in a Hardware Description Language (HDL) such as Verilog and elaborates it into a set of arbitrarily large logic functions. These logic functions are then broken down into smaller functions that each can fit into one single LUT. This process is called technology mapping and is, in the case of VTR, performed utilizing the tool ABC. This step involves a complex optimization problem that, by default, primarily tries to reduce the depth of the resulting technology-mapped network. Moreover, it tries to reduce the overall number of functions [17]. In the next step, the logic functions are grouped into clusters. This so-called packing is performed by the framework Versatile Place and Route (VPR). One cluster is mapped into one instance of a (physical) logic block. For this purpose, VPR already assigns the logic functions to LUTs inside the logic block during the packing stage and also performs the block-internal routing. After the packing step, the clusters are placed on the grid of available physical logic blocks. Simulated Annealing is utilized as a heuristic optimization strategy. After an initial legal placement is found, the clusters are moved across the grid in a way that reduces the overall cost function, with cost estimated through connection length. During Simulated Annealing, temporary cost increases are accepted to allow escape from local minima. After placement, global signals connecting the logic blocks with each other need to be routed through the global interconnect. While routing, the router is allowed to modify the pin assignment of the logic blocks again if needed to achieve an optimal result. The full process is shown in Figure 1.

### 2.3. From Memristor to Memory Cell Defects

For the use case of digital electronics, memristors can be viewed as programmable switches that can be programmed to be in either a Low Resistive State (LRS) or a High Resistive State (HRS). Based on [7], memristor defects that are introduced during manufacturing are categorized as

S@L: Stuck at LRS. This refers to the case where the memristor permanently stays in the LRS.

S@H: Stuck at HRS. This refers to the case where the memristor permanently stays in the HRS.

S@U: Stuck at unknown. This refers to the case where the memristor remains stuck in any possible intermediate state, without changing its value.

**Figure 2:**
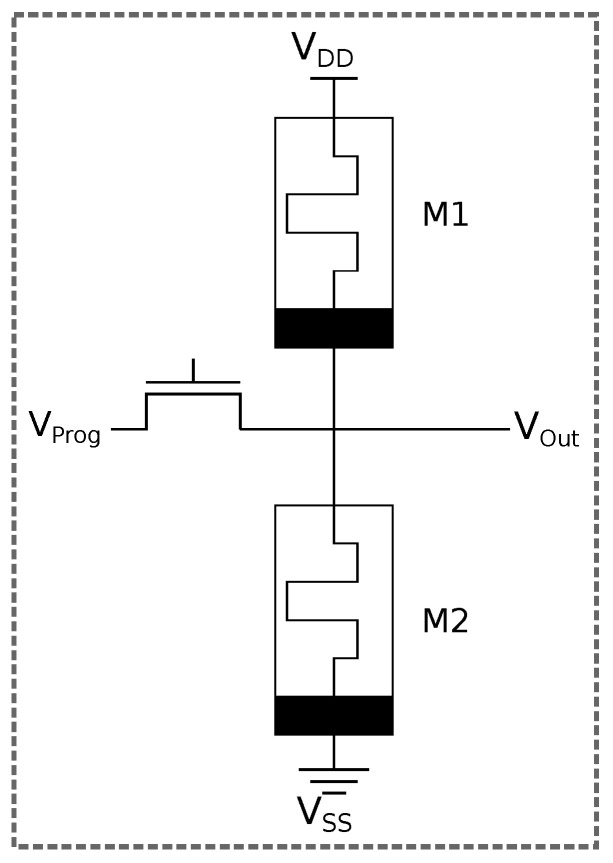
Architecture of a 1T2R memory cell.

Based on this error categorization and the architecture of the memristive LUT memory cell, a defect model on the level of a single cell can be inferred. Figure 2 shows the architecture of a 1-Transistor-2-Resistor (1T2R) memory cell. In this configuration, two memristors are connected in series between VDD and GND. The memristors must be programmed in opposite states to produce a proper output value. This memory cell architecture was chosen because it requires only two memristors per memory cell, without an in-datapath memristor, and the high achievable resistance across the two memristors in either state. This has the added benefit of being fairly resistant to cycle-to-cycle variability of the devices and drifting over time, as the ratio of the memristor resistances is the determining factor instead of a single narrow resistance value. Having only a few memristors per memory cell reduces area consumption and defect vulnerability. Not having an in-datapath memristor is favorable from an electrical perspective in the FPGA use case, where the memristor-based memory cell is essentially constantly read. This constant voltage supply prohibits the use of even smaller memory cells like 1T1R, due to their higher static power consumption and stronger drifting of their state. Assuming a larger FPGA, an amount of memory cells in the magnitude of $10^{5}$ to $10^{6}$ is required. In order to reduce the overall static power consumption to a reasonable level, the voltage dividers in the memory cells need to have a very high resistance at all possible states. Since memory cells do not need to be rewritten at full operating speed of the FPGA, the material properties of the memristors can be tuned to disregard writing speed and energy to some degree and rather yield a maximized memory window with an HRS as large as possible.

### 2.4. Modeling Defective Memory Cells

If only one of the memristors in a 1T2R cell is defective, the other one can still be programmed in such a way that the memory cell can deliver a valid value. However, this value is fixed and determined by the type of defect. The memory cell is therefore Stuck at One (S@1) or Stuck at Zero (S@0). If both memristors are defective, it is possible that they are stuck at the same value and therefore do not deliver a valid output value. The memory cell is therefore Stuck at Undefined (S@U). For this work, it is assumed that an individual memristor is unlikely to be S@U. If one memristor were to be S@U, the entire memory cell would also be S@U and thus would need to be discarded entirely.

**Figure 3:**
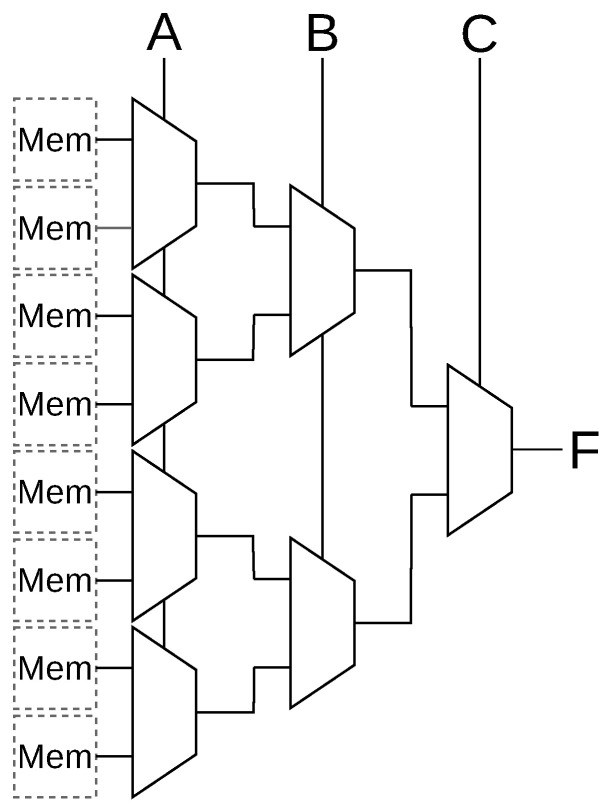
Example 3-Input LUT Architecture with input signals A, B, C and one output F.

**Figure 4:**
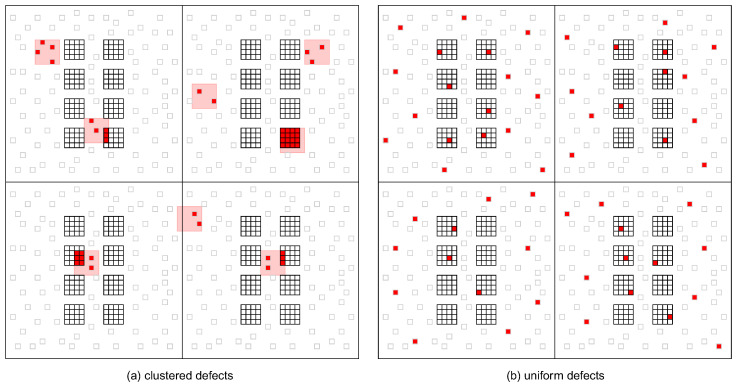
Uniform errors and clustered defects on a two-by-two grid of logic blocks, each with 8 LUTs in the center surrounded by routing memory cells. Defective cells are highlighted in red and clustered defect areas in pink.

Individual memory cells are combined into LUTs as shown for the case of a 3-Input LUT in Figure 3. LUTs can optionally be designed to be fracturable by adding additional output circuitry. Fracturable then means that an N-input LUT can be logically separated into two (N-1)-input LUTs which share some or all of their inputs. This allows for better utilization of LUTs when smaller functions need to be mapped. Fractured LUTs remain a single physical unit sharing memory cells. For this work, we make the assumption that the memory cells within each of the LUTs, fracturable or not, are organized in rectangular groups and placed in the center of a logic block and are surrounded by memory cells that belong to the local and global routing infrastructure, as depicted by Figure 4.

This work mostly considers defects which already manifested during production of the device due to the typical use case of FPGAs, where the configuration memory is programmed once upon startup of the device (or in case of NV-FPGAs, once during deployment) and rarely changed afterwards, requiring few write cycles per memory cell. Erratic bit failures, where single memristors temporarily get stuck for very few cycles only, can partially be compensated for through more resource-intensive feedback-based writing methods that can be adapted from their use in crossbar structures like Write-Verify [18]. Only when a write failure leads to a permanently stuck cell should it be considered as a defect under the scope of this work.

Different fabrication issues can result in various types of defect distributions. Based on the fabrication defects described in [14,15,16], two different defect scenarios can be inferred. While material defects, small contamination, and forming defects are likely to cause single memristors to be defective, particle contaminations with larger particles are more likely to affect whole clusters of memristors simultaneously. Figure 4b shows a uniform defect distribution that affects single memory cells independently. Figure 4a illustrates a clustered defect pattern, where entire groups of memory cells are affected simultaneously. Because both memristors of a cell are likely to be defective in the clustered scenario, the occurrence of S@U defects at the level of memory cells is much more likely.

For measuring the defects in the configuration memory, FPGAs inherently offer a built-in solution. The configuration is written to the memory cells via a register chain. The same register chain can be used to retrieve the outputs of the memory cells, which allows for readback of the actual values including all potential defects present for all configuration memory cells. This eliminates the need for specialized defect measurement techniques or additional measurement hardware. Furthermore, the bitstream coming out of the FPGA allows for spatial classification of the defects as each memory cell bit can be linked to its physical location. This allows for very fine-grained defect pattern analysis on actual devices after manufacturing and even after deployment.

The defect map produced by this readback is the foundation for the fault-tolerant synthesis performed in this work, obtained through a defect classification run of the FPGA and different for each device, as each device has an individual defect distribution. Such a classification consists of programming all memory cells to logical one, reading back the values, then programming all cells to zero and reading back the values. With this technique, all S@X faults can be found. Alternatively, dedicated Design For Test (DFT) approaches would be feasible. However, this classification alone does not eliminate memory cells that switch unreliably. Such defects can only be found after programming with the actual synthesized design. If the readback of the written bitstream differs from the synthesized one, a reprogramming can be conducted and any cells remaining in the incorrect state can be marked as S@U and avoided during any subsequent synthesis the same way fabrication defects are.

### 2.5. Mapping into Defective LUTs

When mapping into *k*-input LUTs that contain defective memory cells, two cases need to be distinguished. In the first case, the LUT contains at least one memory cell that is S@U. Since the value of this memory cell is unknown, it must be ensured that the value of the memory cell is never propagated to the LUT’s output. Looking at the architecture of a LUT as shown previously in Figure 3, one or more LUT inputs can be connected to a fixed value that effectively deactivates the part of the LUT that contains the defective memory cells. This strategy reduces the number of usable inputs (denoted as *u*), but still maintains some functionality.

The second case is the presence of S@1 and S@0 defects. While these defects can be allowed to propagate their value to the output of the LUT, it must be ensured that the value aligns with the value of the function that this specific LUT realizes. This alignment can be achieved in three stages. First, the function may be aligned with the defects to begin with. Second, the inputs of the LUT can be permuted by interchanging two of its inputs. This simple permutation reorders the function’s terms, which can help to align the function with the defects in the LUT. If the simple permutations did not deliver a valid solution, it is furthermore possible to apply a limited number of random permutations by shuffling the inputs. Due to the prohibitive runtime cost, exhaustively attempting all permutations is not feasible for larger designs.

This mapping check needs to be performed during the placement stage of the synthesis process. During this stage, pre-determined groups of logic functions are placed onto the grid of available logic blocks. During the previous grouping of the logic function, a mapping of logic functions to specific LUT instances within the logic block was determined. If no valid mapping could be found with the techniques described above, it is also possible to reassign the logic functions to different LUTs inside the logic block during placement. These changes, as well as the input reassignment of individual LUTs, must be rectified after the placement, and the restrictions implied by this must be propagated to the router that routes the signals connecting the logic blocks. If the reassignment of the LUTs does not help to find a valid mapping, another logic block must be used altogether.

### 2.6. Logic Functions and Their Properties

When considering the mapping of logic functions onto defective hardware, it is crucial to identify the properties of functions that facilitate this process. One major influence on the mappability of a logic function is the number of variables *n* in comparison to the number of LUT inputs *k*. If the function has fewer variables than the LUT has inputs (n<k), the mapping becomes significantly easier, as it is possible to avoid defective memory cells altogether by simply deactivating inputs.

Another factor is the size of the on_set and off_set. The on_set represents all input combinations of the logic function that evaluate to a one, and the off_set represents all input combinations that evaluate to a zero. The terms in the on_set are called minterms, and the terms in the off_set are called maxterms. These two sets are mutually exclusive, and the total number of terms in both sets combined is always $2^{n}$. Suppose a physical LUT contains one S@1 memory cell, and the on_set of the function to be mapped onto it only contains one term. In this case, it is harder to find a permutation that moves the single minterm to the defective memory cell. On the other hand, if the on_set contains many terms, it is easier to find a permutation that fulfills the condition imposed by the defect. Since one set can only grow at the expense of the other, the optimal case is that both sets are equal in size. This idea is formulated as the following metric:

**Definition** **1** (Balance of a logic function).

$$balance=\left\{\begin{array}{cc} \frac{\left|on\_set\right|}{\left|off\_set\right|}, & \mathrm{if}\;|off\_set|>|on\_set|\\ \frac{\left|off\_set\right|}{\left|on\_set\right|}, & \mathrm{else} \end{array}\right.$$

The balance of a logic function describes the difference in size of the function’s on_set and off_set, with a value of one meaning that they are equal in size, and a value of zero meaning that there is only one type of term.

Taking the number of variables and the balance of a function into account, a combined metric can be formulated to describe how difficult it is to map a logic function onto a defective *k*-input LUT:

**Definition** **2** (Mapping hardness of a logic function).

$$hardness=\frac{2^{n}\cdot \left(2-balance\right)}{2^{k+1}}$$

A value of one means that the function is most difficult to map with n=k and a balance value of zero. A small value means that the function is easy to map. The number of variables relative to the number of LUT inputs has an exponential impact on the mapping difficulty, while the balance has a linear impact on the mapping hardness. The different weights given to the two parameters *n* and balance represent the fact that they impact the mappability differently.

This metric is based on empirical studies. When looking at data indicating how many potentially defective LUTs a logic function can be mapped to, it is apparent that the number of variables is the main determining factor. For example, a function with n=k can only reach a certain maximum mappability, and a function with b=k−1 reaches a higher maximum mappability, no matter the balance of the functions. Between these boundaries determined by the number of variables, mappability scales approximately linearly with the function’s balance. When aggregating the mappability of all functions in a design, a good estimation can be provided of how difficult it is to map this design into an FPGA with defective memory cells.

Based on the definition of the mapping hardness of one logic function, the mapping hardness of an entire cluster can be determined by aggregating the individual mapping hardness values of all contained functions and dividing them by the number of available LUTs inside the block:

**Definition** **3** (Mapping hardness of a logic cluster).

$$hardness_{\mathrm{cluster}}=\frac{1}{N}\sum_{i=0}^{\mathrm{functions}\;\mathrm{in}\;\mathrm{cluster}}hardness_{i}$$

### 2.7. Improving the Mapping Results

The most straightforward approach to improve the mapping result is to simply provide more logic blocks than necessary, allowing the placer to use different logic blocks in the case of an unsuccessful mapping. This approach is known as over-provisioning.

However, the discussion about properties of logic functions in Section 2.6 invites more sophisticated methods. The first option is to aim for improving the mappability of individual logic functions. For this purpose, the technology mapping can be modified to consider reducing the mapping hardness as an additional secondary optimization goal. Traditionally, technology mapping tries to reduce the network depth and the network size foremost. To reduce the impact on the timing behavior of the design, it is still necessary to prioritize the depth during the modified technology mapping.

In the case of over-provisioning, simply providing more logic blocks on the device leaves the clusters unchanged and still as fully packed as they would be for smaller devices. In order to leverage the entire available area, the clusters can be packed more sparsely. Instead of simply reducing the number of functions in one cluster, the total mapping hardness of the cluster can be utilized, allowing for more functions in one cluster if they are easier to map and fewer functions if the functions are more challenging to map. For this purpose, an upper mapping hardness limit can be defined. If a cluster would exceed this limit, a new cluster is started. The upper limit can be either statically applied based on a user’s input or dynamically determined based on the defect rate of the device. The factor, called “defectiveness” for brevity, is calculated as an average of the defect rate of the individual logic blocks. Factors that determine the defectiveness of a logic block are the number of usable LUT inputs and the remaining defects that are not already otherwise covered by deactivated inputs. This approach allows for dynamically adjusting the trade-off between using more clusters (and therefore more area consumption) and a better mappability.

## 3. Results

### 3.1. Implementation and Methodology

**Figure 5:**
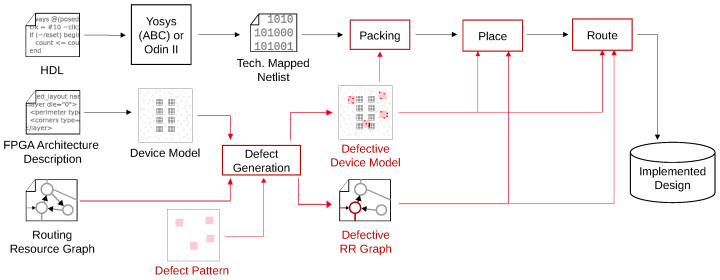
Defect-aware modified VTR Synthesis flow with changes highlighted in red.

To simulate the effect of defective memory cells on an FPGA’s functionality, the VTR toolchain was extended as shown in Figure 5. For this purpose, it was extended with a tool that generates a model of an FPGA architecture at the memory cell level from a given architecture description. The model contains all memory cells belonging to the LUTs and places them in regular groups inside the logic blocks as depicted in Figure 4. Memory cells belonging to the interconnect are not simulated in this paper. However, the impact of fabrication defects on the interconnect is part of ongoing research.

After generating the model, memory defects are applied to it. For this purpose, an algorithm iterates over all memory cells and determines for every cell whether it is defective or not. The probability of a memory cell being defective is determined based on a global defect pattern. The defect probability of a memory cell is derived from the probability of one of the contained memristors being defective. If a memory cell is determined to be faulty, the specific defect value (S@1, S@0, or S@U) is randomly selected based on the chosen fault distribution.

Based on the memory cell model, the tool constructs an updated FPGA model. The model contains all LUTs on the device, along with their exact positions and any defects. Additionally, the LUT model provides functionality for checking whether a given logic function can be mapped into the selected LUT. In addition to determining whether a mapping is possible, the function also returns a permutation, if required, and identifies inputs that need to be fixed to a constant value, if necessary. To check mapping compatibility, the implementation first attempts to deactivate as many inputs as possible to minimize the number of defects and then checks if the function can be mapped without any permutation. If this is not the case, all simple permutations that only swap two inputs are tested. If no solution is found, random permutations are applied. The user can specify the upper limit on the number of random permutations. By default, the use of random permutations is disabled.

This compatibility check was integrated into VPR. During placement, it is performed with candidates being sorted in such a way that the functions always end up in the most defective LUT to which they can be mapped. This ensures that an easy-to-map function does not end up in the least defective LUT, preventing another harder-to-map function from being mapped. If the function cluster cannot be mapped, the current Simulated Annealing move is rejected, and another one is attempted.

As already mentioned, the placement determines which LUT in a logic block a function must be mapped to and how it must be permuted, which in turn affects the logic block’s internal routing. Since this was originally determined during the packing stage and has therefore become invalid, a second packing step must be performed to account for these modifications. During this second packing step, the clusters’ composition remains unchanged. However, the logic block’s internal LUT assignment, the function permutation, and the logic block’s internal routing are rectified to incorporate the changes made during placement. Additionally, the synthesis flow was modified so that the router is not allowed to permute LUT inputs on its own. However, the global routing is allowed to change the logic block’s input assignments, as long as this does not affect the LUT’s input assignment. This can be ensured by providing a full crossbar for the logic block’s internal routing.

### 3.2. FPGA Architecture and Benchmarks

To evaluate the defect-aware synthesis flow, the results for four different FPGA architectures are compared. These architectures are all based on the k6_frac_N10_40nm architecture described in [19]. Each architecture is comprised of a grid of logic blocks. The perimeter hosts Input/Output (IO) blocks, and the corner blocks remain empty. Some of the evaluated benchmarks are very IO-heavy, and therefore, simple IO blocks, only containing one IO pad, would have been the limiting resource, inflating the required device size. Therefore, the number of pads per IO block was increased to fit the required number of IOs.

For the evaluation, logic blocks were varied in terms of the number of LUTs per logic block and the number of LUT inputs. The parameters of all four architectures are presented in Table 1. All logic blocks contain the same number of LUTs and all LUT outputs can be (optionally) stored in a register. Instead of connecting all LUT inputs to the inputs of the logic block, only 2/3 of them are, as is done in the k6_frac_N10_40nm architecture. All LUTs, respectively register, outputs are connected to the outputs of the logic block containing them. To allow free interchangeability of LUT inputs, as utilized by the defect-aware synthesis, a full crossbar topology was chosen for the intra-logic-block interconnect. Each logic block has its input pins connected to 15% of the global interconnect segments (fc_in = 0.15) and its output pins are connected to 10% (fc_out = 0.1). The length of the interconnect segments is 4.

The minimum grid size of the architecture was determined by using the automatic sizing functionality of VPR on an error-free FPGA. The size was over-provisioned to be twice the minimum required size to accommodate for congestion in routing. The channel width of the global interconnect was set to 1.2 times the minimum value determined by VPR.

The MCNC20 benchmark suite [20] was chosen for this evaluation. Although the MCNC20 benchmark set primarily consists of smaller designs that are not always representative of modern use cases, the advantage is that they can be implemented as soft-logic-only designs. Other, more modern benchmark suites heavily rely on hard blocks, which are out of scope of this work. However, when synthesizing such designs into soft-logic only, it is possible to apply the presented approach as well. This was done with additional benchmarks from the VTR Benchmark suite [8] for verification. As these designs behave sufficiently similarly to the ones obtained from the MCNC20 benchmark suite, they are omitted here.

The mapping results are presented in terms of the number of designs that can be mapped successfully onto the defective device and in terms of the average increase of the critical path length compared to the error-free case. Looking at the (relative) increase of the critical path length abstracts the fact that every design has a different critical path length for every architecture, allowing for robust conclusions about the impact of defects on mapping quality.

### 3.3. Defect Pattern Types

For this work, two distinct types of defect distributions are distinguished: uniform and clustered defects. The cluster defect size is chosen based on a particle contamination with particle sizes that are allowed in clean rooms according to the ISO norm ISO 14644-1 [21]. While clean rooms of grades 1 to 3 do not allow particles larger than 1 μm, grades 4 and 5, which are typical for university chip labs, only allow very few particles larger than 1 μm. For this work, we simulated particle sizes ranging from 0.1 μm to 5.0 μm. For brevity and where not otherwise noted, we show the results for clustered error distribution assuming a particle size of 1 μm. Assuming a 28 nm process node with $4F^{2}$ memristor size, each such particle can, in the worst case, cause a cluster of 12 by 12 memory cells to be defective.

These defect distributions are based on reasonable assumptions for the defect patterns (random and particle contamination patches). However, since every fabrication process is different, the underlying software framework allows for arbitrary defect pattern generation. As soon as reliable data on the manufacturing capabilities of a specific BEOL process is available, the simulations can be adapted and rerun with fitting defect patterns and rates.

The assumed defect rates were chosen based on the expected defect probability during BEOL manufacturing, which traditionally is higher than that of regular manufacturing, and the resulting approximation of what overall percentage of technology elements could possibly be affected. Specific error rates of commercial memristor processes are hard to establish and usually not released publicly. Based on private discussions with material suppliers, we expect defect rates above 1% for mass-produced memristors in non-crossbar structures. The first approximate analysis has shown that the therefore assumed defect rates between 1 % and 5 % will affect a significant number of technology elements, and thus an operation without tailored countermeasures is not possible. Much lower defect rates on the order of 0.05% as they might be seen in more mature technology processes will at most affect one eighth of the total technology elements. In FPGA synthesis, this magnitude of LUTs can be covered through over-provisioning, as resource limitations in, e.g., the routing network restrict the percentage of LUTs that can be utilized at most in a specific design anyway. When assuming such low defect rates, the simple approach of just deactivating the affected technology elements could already be sufficient to fully map a given design onto such an FPGA. Therefore, a more detailed analysis and defect mitigation are only covered for the comparatively higher defect rates of BEOL manufacturing.

For the same overall number of defective memristors, the two defect patterns exhibit very different impacts on the results. For uniform defect patterns, the number of affected memory cells is close to the number of defective memristors, since in most cases, only one memristor per memory cell is defective. Therefore, the primary concern regarding the uniform defect pattern is the sheer number of memory cells that are S@0 or S@1. On the other hand, the clustered defect pattern likely affects both memristors in a memory cell simultaneously, and therefore, the number of affected memory cells is only half as big. The primary concern regarding the clustered defect pattern as a result is the presence of S@U defects that require deactivating an input.

**Figure 6:**
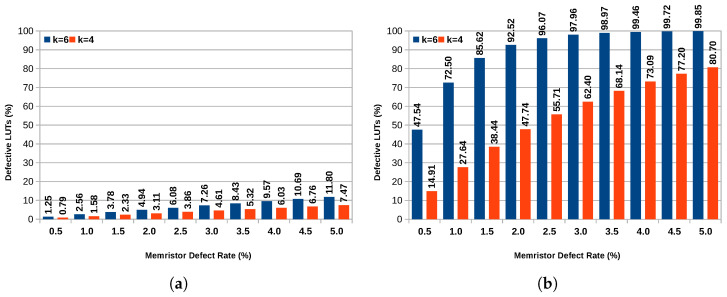
Percentage of 4- and 6-Input LUTs that contain at least one defective memory cell for different defect distributions. (**a**) Clustered Distribution; (**b**) Uniform Distribution.

The number of LUTs that contain at least one defective memory cell is significantly larger for the uniform defect pattern in comparison to the clustered defect pattern, as can be seen in Figure 6.

Assuming a uniform error pattern and 6-input LUTs, almost all LUTs contain defective memory cells for memristor defect rates of more than 3%. The number of affected LUTs is significantly smaller for the clustered defect pattern and also for 4-input LUTs. These results show that the clustered defect pattern has a less severe impact on the device. Moreover, it shows that 4-input LUTs are likely the superior choice in terms of defect tolerance. These findings also show that the approach of simply discarding entire slices if they are defective, as proposed in [12], will not be sufficient when dealing with memory cell defects.

Additionally, an investigation into much smaller error probabilities, ranging from 0.01% to 0.05%, was conducted to allow comparison with other defect-tolerant mapping approaches that are more focused on defects that occur during CMOS fabrication for small structure sizes. This defect rate corresponds to 100 to 500 defective memristors per million, as may be observed in mature manufacturing processes. The LUT defect rate for 6-input LUTs varies from 0.03% to 0.13% for the clustered defect distribution and 1.29% to 6.23% for the uniform defect distribution. For 4-input LUTs, the LUT defect rates range from 0.01% to 0.07% and from 0.34% to 1.61%, respectively.

The difference in the impact of the defect patterns is also evident in the mapping results shown in the next section. The uniform defect pattern degrades results significantly more heavily than the clustered defect pattern does for the same overall number of defective memristors.

### 3.4. Mapping Hardness

**Figure 7:**
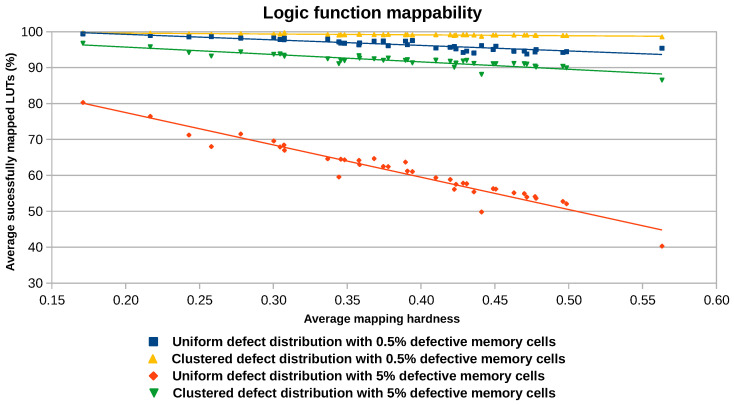
Average mapping hardness of a design in comparison to the average percentage of LUTs the design’s logic functions could successfully be mapped to for 6-input LUTs.

The mapping hardness metric was introduced to measure the difficulty of mapping a logic function onto a defective LUT. Since the mapping success depends on the random distribution of defects inside each specific LUT, the mappability is determined as an average. For this purpose, the average mapping hardness of all logic functions in each design across all MCNC20 benchmarks was determined for two different technology mappings, yielding 40 designs with distinct average mapping hardness values. Additionally, a huge FPGA with more than 33.000 potentially defective LUTs was generated. For all logic functions, a mapping into all LUTs available on the device was attempted, and the average number of successful mappings for the individual benchmarks was determined. Figure 7 plots the average mapping hardness against the average percentage of LUTs the logic functions could be mapped onto. The average number of LUTs a function can be mapped to successfully is a good proxy for determining the mappability of a logic function.

The plot features four different defect patterns: two uniform defect distributions with 0.5% and 5% error rates, as well as two clustered distributions with patches of six by six defective memory cells and error rates of 0.5% and 5%. There is a strong linear correlation between the mapping hardness and the average number of LUTs the logic functions could be mapped to successfully. Additional simulations show that the same is true for 4-input LUTs. These results validate the mapping hardness metric as a reliable proxy for estimating the difficulty of mapping a design onto a defective FPGA, making it suitable for synthesis optimization.

### 3.5. Architecture Comparison

**Figure 8:**
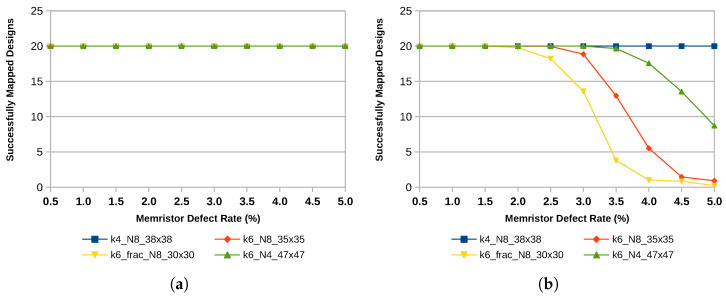
Successfully mapped designs of the MCNC20 benchmark suite for different architectures. (**a**) Clustered Distribution; (**b**) Uniform Distribution.

Architecture choices, such as the size of the LUTs, whether or not they are fracturable, and the size of logic blocks, impact the fault tolerance of the device. Currently, the trend in technology is leading towards larger LUTs with more advanced options for fracturability. The results of this study show that for fault tolerance, smaller, non-fracturable LUTs are the better choice. Figure 8 shows the number of successfully mapped designs. It can be observed that for an architecture with 4-input LUTs and a uniform error distribution, 20 out of 20 designs can be mapped successfully even at high error rates between 2% and 5%. In contrast, the number of successfully mapped designs decreases rapidly as the defect probability increases in the case of 6-input LUTs. Reducing the number of LUTs within one logic block can help improve the results, while fracturable LUTs further reduce the number of successfully mapped designs as inputs of fractured LUTs remain shared after the split.

**Figure 9:**
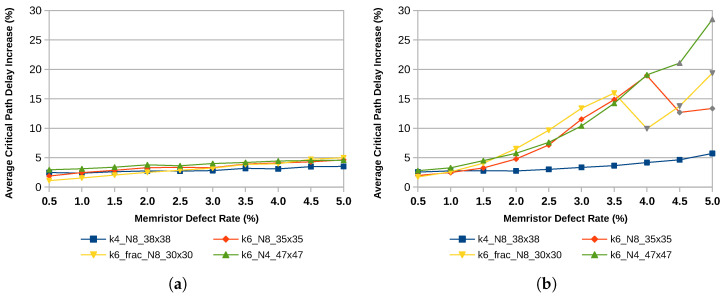
Average critical path increase from the error-free case of the MCNC20 benchmark suite for different architectures. Cases where mapping failures lead to the false appearance of improvement are marked in gray. (**a**) Clustered Distribution; (**b**) Uniform Distribution.

The same findings hold for the average critical path increase shown in Figure 9. For large defect rates and a uniform defect pattern, only a few designs can be mapped successfully and therefore contribute to the average critical path increase. This is why some data points, gray in this case, start to deviate from the overall trend line. Nevertheless, 4-input LUTs exhibit a smaller increase in the critical path delay compared to 6-input LUTs.

We also investigated defect probabilities ranging from 0.01% to 0.05%. As expected, all designs can be mapped successfully across all architectures, with an average critical path increase in the sub-3% regime.

The resource usage in terms of logic block utilization is depicted in Table 2. To compare the size of the architecture relative to chip size, the last column lists the average number of memory cells required to implement the design’s logic. This includes all memory cells that belong to the LUTs inside a used logic block. The architecture with 4-input LUTs requires the least number of logic memory cells. The reason for this behavior is the technology mapping. The technology mapping was performed using the if command implemented in ABC. For 4-input LUTs, the technology mapping yields denser results than it does for 6-input LUTs. This can be observed when examining the average number of variables in logic functions after technology mapping. When mapping into 6-input LUTs, the average number of variables per logic function is only 4.45, with only 31.6% of the functions featuring six variables. When mapping into 4-input LUTs, the average number of variables per logic function is 3.33, with 53% of the functions actually having four variables.

### 3.6. Improvements

In this work, we present three strategies to improve mapping quality beyond the basic approach. The first strategy is to, in addition to the simple permutations, allow up to a set number of random permutations. For this improvement, the number of allowed random permutations was initially set to 100, to balance mapping success with runtime cost. The second strategy is to incorporate the mapping hardness as a secondary optimization goal during technology mapping. The third strategy is to limit the cluster’s complexity by starting a new cluster when adding another function to the old cluster would exceed a defined upper limit for accumulated mapping hardness. This upper limit is determined based on the device’s defectiveness.

**Figure 10:**
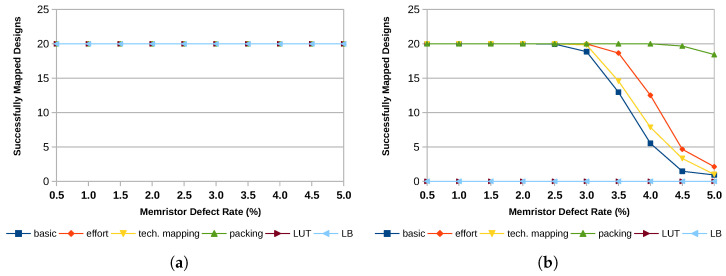
Successfully mapped designs of the MCNC20 benchmark suite with mapping improvements in comparison to the basic mapping and deactivating entire LUTs and logic blocks (LB) for the architecture k6_N8_35x35. (**a**) Clustered Distribution; (**b**) Uniform Distribution.

**Figure 11:**
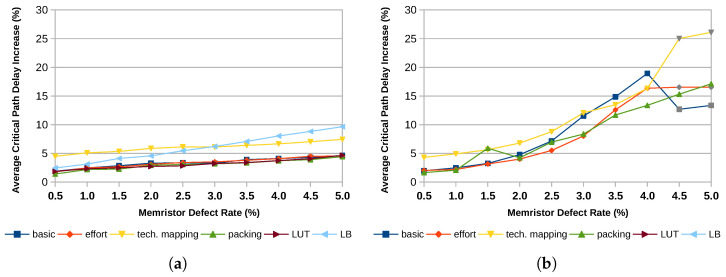
Average critical path delay increase from the error-free case of the MCNC20 benchmark suite with mapping improvements in comparison to the basic mapping and deactivating entire LUTs and logic blocks (LB) for the architecture k6_N8_35x35. Points for LUT and LB are omitted where mapping failed. Cases where mapping failures lead to the false appearance of improvement are marked in gray. (**a**) Clustered Distribution; (**b**) Uniform Distribution.

Figure 10 shows the results for all three improvements in comparison to the non-improved basic mapping (dark blue line) for the 6-input LUT architecture with 8 LUTs per logic block. Additionally, the plots feature two data lines showing the performance of two baseline approaches taken from the literature. These approaches deactivate the entire LUT or the entire logic block in the presence of an error. The improved packing can achieve the most significant improvement. While allowing random permutations also yields some improvement, the enhanced technology mapping does not significantly enhance the number of mappable designs. This becomes even clearer when looking at the average critical path increase in Figure 11. While all other improvements can also improve the critical path delay, the modified technology mapping with a secondary optimization goal performs worse for almost all defect patterns.

The size of the FPGA was set to twice the size required by the largest design. This results in a peak logic block utilization of roughly 45% for all architectures and an average logic block utilization of roughly 20%. Allowing additional random permutations does not change the number of clusters and, therefore, does not change the logic block utilization.

The modified technology mapping, on the other hand, increases the number of logic functions. The mean number of functions per design increases by about 13% compared to the default technology mapping, which also leads to an increase in the number of clusters. However, except for one design, the depth of the resulting technology-mapped designs remains unchanged.

Since the upper mapping hardness limit for the improved cluster packing is determined dynamically, the increase in clusters depends on the defect pattern. For patterns where all designs can already be mapped without redistributing functions to reduce the cluster’s mapping hardness, the number of clusters remains unchanged. For the other defect patterns, the number of clusters grows rapidly, utilizing up to twice as many clusters for the uniform 5% case.

### 3.7. Comparison to Other Approaches

As discussed in Section 2.1, most approaches to fault tolerance in FPGAs avoid using defective elements altogether. Their granularity ranges from individual LUTs to entire slices. Since larger areas are more likely to contain at least one defective memory cell, this approach quickly becomes infeasible for high defect rates. In order to compare the approach presented here, we evaluated the mapping results for a fault-tolerant approach that avoids LUTs containing defective memory cells and one that avoids entire logic blocks in the presence of defective memory cells. The results presented here are from the 6-input LUT architecture with 35 by 35 logic blocks. The results are visualized in Figure 10 and Figure 11 and labeled as LUT for the LUT-avoidance approach and LB for the approach operating on logic block level.

When avoiding defective LUTs, the mapping becomes impossible for uniform defect patterns if the error probability increases above 0.5%. For smaller defect probabilities from 0.01% to 0.05%, the mapping can be completed successfully. However, the average increase of the critical path length is slightly higher, up to 3.7% for the 0.05% defect case. For clustered defect distributions, all 20 designs could be mapped successfully, even for a 5% defect rate. The average increase of the critical path length is comparable to that of the approach presented in this work and falls in the sub-5% regime.

The main issue with the strategy of avoiding defective logic blocks is the sheer number of them. For uniformly distributed defects, all logic blocks are defective for a memristor defect probability above 1%. Even smaller defect probabilities of 0.01% to 0.05% result in 9.9% to 40% defective logic blocks. For the clustered defect distributions, the numbers are slightly better, with 5.3% to 48.9% defective logic blocks at memristor defect rates of 0.5% to 5%. This also reflects on the mapping result. While the designs could have been mapped successfully for defect rates of 0.01% to 0.05% and assuming a uniform defect distribution, this is not the case for larger defect probabilities. The average increase of the critical path length is also larger, ranging from 3.1% at a 0.01% defect rate to 7.8% at a 0.05% defect rate.

Because of the overprovisioning of the tested architecture, all designs can still be mapped successfully for the analyzed clustered error patterns by just avoiding the defective elements. However, this comes at the cost of an increased critical path that is up to 9.7% longer, nearly twice as much as for the proposed approach, and the necessity of providing more than twice as many logic blocks as required for the design to allow enough headroom for the avoidance.

## 4. Discussion

Evaluation results clearly show that the number of defective LUTs is quite significant, even for small memristor defect rates and especially in the case of a uniform defect distribution. Previously discussed approaches that involve deactivating entire LUTs or even slices are therefore not a viable solution in such a scenario. More sophisticated fault tolerance mechanisms are required. However, the pattern of the physical defects is the largest factor in determining the degree of defectiveness of the overall device. While uniform defects affect a large number of LUTs, clustered defects hit fewer LUTs but damage them more severely.

### 4.1. Effects of Architecture Changes

The presented results clearly show that 4-input LUTs are more defect-tolerant than 6-input LUTs. LUT defect severity depends on both the number of compromised memory cells and the extent of the damage caused by each defect. While the number of defective memory cells scales linearly with the capacity of the LUT, the number of required memory cells grows exponentially relative to the number of LUT inputs. Conversely, the impact of a single defect diminishes only linearly as the number of inputs increases. Because this exponential growth in memory cell count far outweighs the linear decrease in impact of each defect, larger LUTs are more affected by defects than smaller ones. Another effect in favor of smaller LUTs is the likelihood of LUTs containing defective memory cells at all. Because of the exponentially smaller size of smaller LUTs, far fewer LUTs are defective to begin with. This allows for easier avoidance of the defective LUTs altogether. A comparable effect takes place when considering differently sized logic blocks. It is weaker in this case, as the number of memory cells grows only linearly with the addition of another LUT. However, the impact of each defect does not diminish with the same factor, again, favoring smaller logic blocks.

Synthesis results also indicate that non-fracturable LUT architectures outperform architectures with fracturable LUTs. This can be explained by the placement of logic functions inside the LUTs. If LUTs are fracturable, logic functions with fewer variables than LUT inputs are always placed in only one half of the LUT. While this behavior is reasonable during packing, since it easily allows adding a second function to the LUT, it reduces the number of inputs that can be deactivated to mitigate defects and therefore deteriorates the mapping result.

These findings clearly contradict the current general trend leading towards larger LUTs, larger logic blocks, and more complex fracturability. This trend is driven by the desire to increase performance by reducing the critical path length. Architectures with larger LUTs and logic blocks generally exhibit better performance in terms of the critical path delay. However, when considering defective devices, the primary concern is to ensure that the design remains mappable. Furthermore, the greater performance degradation of larger LUTs and logic blocks for more severe defect patterns can outweigh the smaller penalty in critical path length for the error-free case, making smaller LUTs and logic blocks the superior choice when defects can be present.

### 4.2. Discussion of Improvements

Among the proposed synthesis flow improvements, the enhanced packing strategy is the most effective. It is capable of increasing the number of successfully mapped designs significantly while also minimizing the increase in critical path length. Moreover, it only requires additional resources if necessary, which enables the deployment of this strategy without incurring unnecessary area expenses.

The same is true for allowing additional random permutations. While the benefit is not as significant as that of the improved packing, attempting additional permutations can effectively trade off the mapping tool’s runtime for a better mapping result when there is actual room for improvement. This room for improvement is only present if the dominating defects are the S@1 and S@0 defects, as is the case for uniform defect patterns. When deactivating inputs can mitigate most defects, as is the case for clustered defect patterns that are primarily dominated by S@U defects, allowing more random permutations does not improve results, but also does not require additional runtime.

In contrast, the modified technology mapping with a secondary optimization goal yields no significant improvement and degrades performance in some cases. In fact, an increase in critical path length can be observed for nearly all defect patterns. The reason for that effect is the comparable slight improvement in mapping hardness, with the average mapping hardness of all designs improving from 0.41 to 0.31, which is outweighed by the increase in the number of logic functions. This leads to the creation of more clusters, which in turn increases the logic depth and thus the length of the critical path. However, further investigation has shown that for scenarios with a high uniform defect probability, combining the modified technology mapping approach with enhanced packing yields a reduction in the number of clusters needed. This shows that there are still some use cases for this strategy. Further research is required to reduce the number of functions while simultaneously reducing the mapping hardness, which would, in turn, allow for better mapping results.

### 4.3. Future Work

The next obvious step is to model and simulate memory cell defects in the interconnect. However, based on findings in [7], it can be expected that the defect tolerance of common interconnect architectures is low. Therefore, more research into defect-tolerant interconnect hardware is required first. With the help of more defect-tolerant routing architectures and intelligent fault-tolerant mapping solutions, the impact of defective routing memory cells can hopefully be minimized. Moreover, over-provisioning the routing resources by adding additional lines and connection points can further help to reduce the impact of defects. This over-provisioning can also be considered cheap in light of the dramatic reduction of area consumption caused by the switch from SRAM cells to memristors, used either as switches or in memory cells.

In the presented approach, geometrical information about error impacts is a side product that is not yet fully utilized. It is feasible to consider using defect information about neighboring sensitive structures to judge whether the defect distribution is more clustered or more uniform and, as a result, derive insights into potential issues of the fabrication process. Additionally, for architectures with multiple LUT-sizes, which, while not exactly common, do exist commercially (e.g., the Gowin GW3A family [22] with both 4- and 6-Input LUTs), the more sensitive LUTs could be used as a sorting criterion during mapping to estimate the likelihood of further defects in the same logic block.

## 5. Conclusions

This paper introduces an approach to map logic functions into defective LUTs in order to deal with high defect rates. The presented evaluation underlines the necessity of such an approach for imperfect devices. The high defect rates of LUTs render traditional approaches that avoid defective elements altogether impractical.

This paper presents a comprehensive analysis of how different FPGA architecture decisions influence resilience to memory cell defects caused by faulty memristors. The results show that, contrary to the industrial trend for SRAM-based FPGAs, smaller LUTs that are non-fracturable in smaller logic blocks exhibit greater defect tolerance.

Furthermore, this work presents different approaches to improve the mapping results. For this purpose, a new property of logic functions, called the mapping hardness, was introduced. The evaluation shows that this metric predicts how well a design can be mapped onto a defective device. Two improvements to the mapping process based on this metric were introduced. Reducing the mapping complexity of entire clusters during packing yielded greatly improved results and made it possible to maintain functionality even for scenarios in which nearly all LUTs exhibit defects.

As a next step, the authors are currently preparing a tape-out of a test chip, where multiple LUT implementations can be evaluated with memristors manufactured as back-end-of-line in-house using different material technologies.

## Figures and Tables

**Table 1:** Properties of the investigated architectures (k: LUT inputs, N: LUTs per logic block).

Architecture	k	N	Logic Block Input Pins	Device Size
k6_N8_35x35	6	8	22	35 × 35
k4_N8_38x38	4	8	22	38 × 38
k6_frac_N8_30x30	6 (fracturable)	8	22	30 × 30
k6_N4_47x47	6	4	16	47 × 47

**Table 2:** Average resource usage of the investigated architectures. The logic memory cells denote all memory cells that belong to LUTs of occupied logic blocks.

Architecture	Average Logic Blocks	Average Logic Block Utilization	Average Logic Memory Cells
k6_N8_25x25	202.1	18.55%	103,475.2
k4_N4_38x38	260.95	20.2%	33,401.6
k6_frac_N8_30x30	176.65	22.6%	90,444.8
k6_N4_47x47	397.75	19.85%	101,824

## Data Availability

The original data presented in the study is openly available at https://tudatalib.ulb.tu-darmstadt.de/handle/tudatalib/5044.2 (accessed on 20 March 2026).

## Abbreviations

The following abbreviations are used in this manuscript:

1T2R	1-Transistor-2-Resistor
ASIC	Application Specific Integrated Circuit
BEOL	Back-End-Of-Line
CAD	Computer Aided Design
DFT	Design For Test
FPGA	Field Programmable Gate Array
HDL	Hardware Description Language
HRS	High Resistive State
IO	Input/Output (block)
LB	Logic Block
LRS	Low Resistive State
LUT	Look Up Table
S@	Stuck at Fault
S@1	Stuck at One
S@0	Stuck at Zero
S@H	Stuck at High Resistive State
S@L	Stuck at Low Resistive State
S@U	Stuck at Undefined
SRAM	Static Random Access Memory
VPR	Versatile Place and Route
VTR	Verilog to Routing
